# 92 ASCS Treatment Impact on Length of Stay Data and Costs for Patients with Small Burns

**DOI:** 10.1093/jbcr/irac012.095

**Published:** 2022-03-23

**Authors:** Jeffrey E Carter, Joshua S Carson, Lisa Rae, Syed F Saquib, Lucy Wibbenmeyer, William L Hickerson

**Affiliations:** University Medical Center- New Orleans, New Orleans, Louisiana; Loyola University Medical Center, Oak Park, Illinois; Temple University, Philadelphia, Pennsylvania; Kirk Kerkorian School of Medicine at UNLV, Las Vegas, Nevada; University of Iowa MD, FACS, Iowa City, Iowa; Spectral MD, Avita Medical, AccessPro Med, Memphis, Tennessee

## Abstract

**Introduction:**

Introduction: Small burns with a total body surface area (TBSA) of < 20% account for the large majority (92%) of burn injury hospital admissions. Autologous skin cell suspension (ASCS) is a novel treatment for acute thermal burn injuries – including small burns -- that is associated with significantly lower donor skin requirements than split-thickness skin grafts, the traditional standard of care (SOC). The ASCS treatment indication was recently expanded from adult patients to include pediatric patients. Previously modeled analyses suggested that ASCS use is associated with a lower hospital length of stay (LOS) and costs savings versus SOC. This study evaluated whether real-world data (RWD) corroborate these findings in small burns and in both adult and pediatric populations.

**Methods:**

Methods: Data were collected from January 2019 through August 2020 from 500 facilities in the United States. Adult patients (age ≥ 21) and pediatric patients (< age 21) receiving inpatient burn treatment with ASCS were identified and matched to patients receiving SOC based on sex, age, TBSA < 20%, and comorbidities. Based on typical BEACON model outcomes, LOS was assumed to account for 70% of total costs and was used as a proxy to assess the data. LOS was assumed to cost $7,554 per day. Mean LOS and costs were calculated for the ASCS and SOC adult and pediatric cohorts. The incremental revenue associated with changes in inpatient capacity was also analyzed.

**Results:**

Results: A total of 151 ASCS and 2,243 SOC adult cases and 19 ASCS and 341 SOC pediatric cases were identified. In adults, the SOC cohort had a higher percentage of patients with TBSA < 20% than the ASCS cohort (82.9% vs. 55.0%). For small burns, sixty-three matches were made for each adult cohort, and seven matches were made for each pediatric cohort. For adults, LOS was 18.5 days with ASCS use and 20.6 days with SOC use (difference: 2.1 days [10.2%]). For pediatrics, the ASCS LOS was 18.6 days, and the SOC LOS was 21.4 days (difference: 2.9 days [15.4%]). This difference led to cost savings of $15,587.62 per adult ASCS patient. Total cost savings with ASCS adult patients were $22,268.03 per patient. The reduced LOS with ASCS adult patients resulted in an increased capacity of 2.0 inpatients per bed per year, which was estimated to increase hospital revenue by $83,894 per burn unit bed annually. Pediatric cost results and savings were similar.

**Conclusions:**

Conclusion: This RWD analysis shows that small burn treatment with ASCS is associated with reduced LOS and substantial cost savings compared with SOC in both adult and pediatric populations, supporting the validity of previous model projections. ASCS use may also significantly increase hospital revenue related to increased inpatient capacity.

